# From Life in the Sea to the Clinic: The Marine Drugs Approved and under Clinical Trial

**DOI:** 10.3390/life11121390

**Published:** 2021-12-11

**Authors:** Emiliano Cappello, Paola Nieri

**Affiliations:** 1 Department of Clinical and Experimental Medicine, University of Pisa, 56126 Pisa, Italy; 2 Department of Pharmacy, University of Pisa, 56126 Pisa, Italy; paola.nieri@unipi.it; 3 Interdepartmental Center of Marine Pharmacology (*MARinePHARMA*), University of Pisa, 56126 Pisa, Italy

**Keywords:** marine drugs, sea pharmaceuticals, EMA, FDA, clinical trials

## Abstract

In the last decades Blue Growth policy in european and non-european countries produced a great impulse in applied marine sciences, comprehending the research of new bioactive molecules in marine organisms. These organisms are a great source of natural compounds with unique features resulting from the huge variability of marine habitats and species living in them. Most of the marine compounds in use and in clinical trials are drugs for cancer therapy and many of them are conjugated to antibody to form antibody-drug conjugates (ADCs). Severe pain, viral infections, hypertriglyceridemia, obesity, Alzheimer’s and other CNS diseases are further target conditions for these pharmaceuticals. This review summarizes the state-of-the-art marine drugs focusing on the most successful results in the fast expanding field of marine pharmacology.

## 1. Introduction

The terrestrial environment of our planet has always been a great source of molecules for the discovery of new drugs and lead compounds. The development of pharmacological resistance to traditional therapies and the difficulties in the treatment of many diseases has broadened the horizon of pharmacological research considering biologically active compounds derived from marine organisms. This was possible thanks to the development of advanced technologies to explore underwater environments that have allowed access to organisms previously unreachable, to the aquaculture techniques and the Blue Economy politics sustaining the expansion of the Blue Growth. A key role was also played by the development of analytical spectroscopy methods, and improved genome sequencing techniques [1,2].

The oceans occupy more than 70% of the Earth’s surface and represent one of the richest components of the Earth’s biosphere. The oceans contain more than 80% of the world’s vegetable and animal species, in addition to an incredible number of microorganisms. The conditions of marine environments are incredibly vast; for example, the temperature ranges from −1.5 °C in the frozen seas of both poles to 350 °C in the hydrothermal ecosystems found on the ocean floor. This is the reason for a extraordinary great biodiversity reflecting a just as big chemodiversity, often very different from terrestrial one [3]. Many chemical classes are represented, such as polyketides, terpenes, saponins, carbohydrates, aliphatic compounds, amino acids, alkaloids, peptides, lipopeptides, proteins and steroidal, triterpene or aliphatic compounds [3]. A very recent review focused on the chemical structures of marine-derived compounds in drug research has been published by Lu et al. [4].

Many bioactive molecules are often of microbial origin, often produced by bacteria or cyanobacteria in symbiosis with higher organisms from which these molecules were isolated [5,6].

The first marine compound used in therapy was the polysaccharide alginate, discovered in 1881 from kelp. Its use as sodium salt (sodium alginate) is for the gastro-esophageal reflux disease since after forming a gel in the gastric lumen it is able to protect the esophageal mucosa from acid [7]. However, a greater research on marine-derived compounds began only in the 1940s, when spongosins were extracted from the sponge *Tethya* or *Cryptotethya crypta*. [6]. Moreover, in 1948, the discovery of cephalosporin C, produced by the fungus *Acremonium chrysogenum*, isolated from seawater samples in Sardinia (Italy), was the starting point for the development of the antibiotic class of cephalosporins [8].

The present review is focused on the marine origin and the pharmacological features of drugs that obtained approval on the market by EMA and/or FDA authorities, and on ongoing clinical trials on sea-derived molecules.

## 2. Marketed Marine Drugs

**Figure 1:**
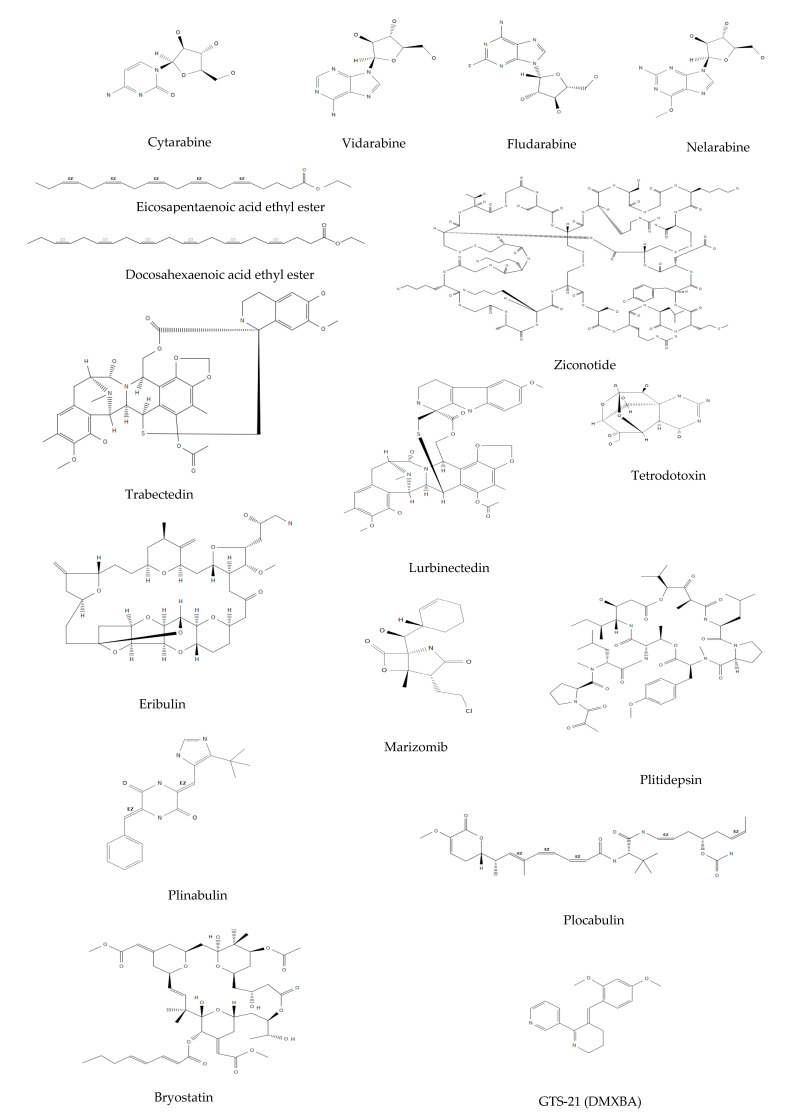
Chemical structures of marine drugs approved or under clinical trials, not including those present in antibody drug conjugates (ADCs) which are reported in Figure 2.

**Figure 2:**
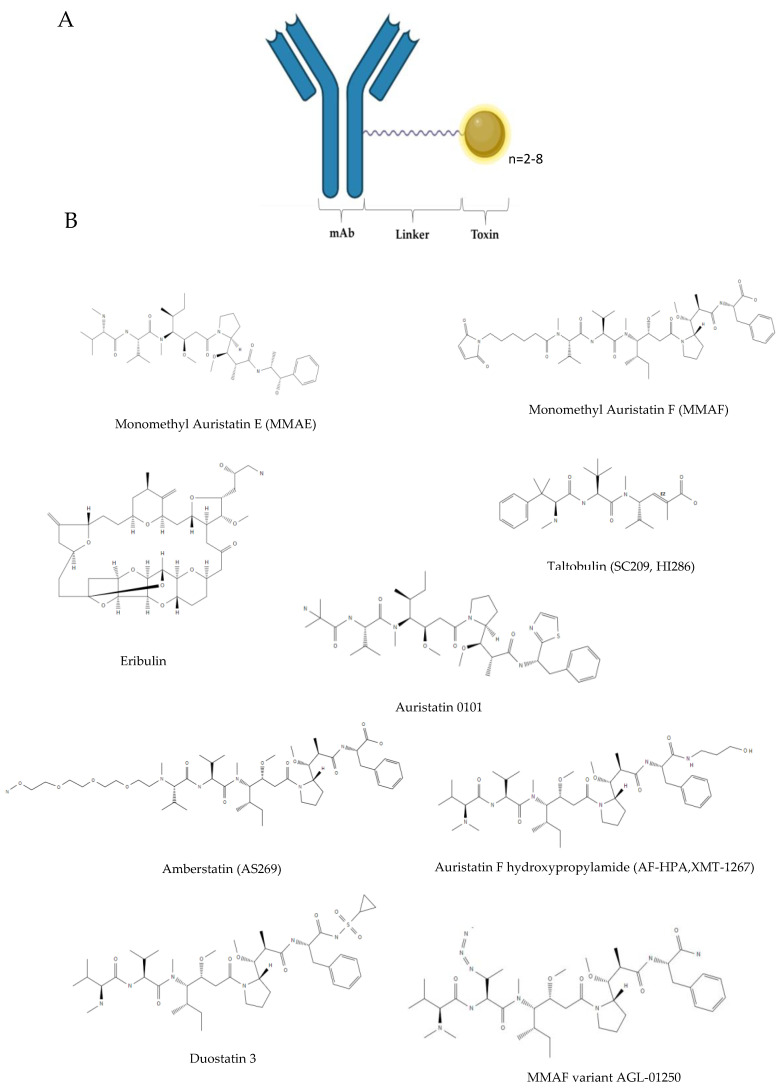
(**A**) General structure of Antibody-Drug Conjugates (ADCs) approved and in clinical trials and (**B**) chemical structures of toxins.

Currently, sea-derived drugs arrived on the EU and/or the USA pharmaceutical market are thirteen (Table 1) of which four received approval in the last three years. They are mostly anticancer agents (ten out of thirteen); however chronic pain, hypertrigliceridemia and viral infections also represent approved uses. In the following paragraphs, the main features of drugs arrived on the market are presented, following the time order of marketing approval (the same of Table 1). All the chemical structures of marketed marine compounds are reported in Figure 1 and Figure 2.

### 2.1. Cytarabine

Nucleosides, the glycosides of purines and pyrimidines, and their nucleotidic phosphate derivatives, are essential components in all living cells playing a relevant role in many biological processes. 

On the other hand, antimetabolite nucleosides are used from the same producer organisms/microrganisms to inhibit the growth of competitors or predators. Antimetabolites are, in fact, similar to naturally produced metabolites, but they alter cell metabolism failing to perform the normal function. Nucleosidic antimetabolites may interfere seriously with normal cell function producing inability to proliferate. 

The discovery of spongosines, D-arabinose nucleosides from *Tethya* or *Cryptotethya crypta* sponge, opened the era of marine drugs and of antimetabolite nucleosidic drugs, giving great opportunity to the anticancer and the antiviral therapy. The *Tethya* or *Cryptotethya crypta* sponge species, widely distributed in the Caribbean Sea, from a taxonomic point of view belongs to the class Desmospongiae of the vast and ancient Phylum Porifera. Among marine invertebrates, Porifera represent the richest phylum in active metabolites, with an average of more than 200 new molecules isolated every year. Accordingly to the role of antimetabolites, the production of cytotoxic agents such as spongouridine and spongothymidine provides a protective role against pathogens, predators and parasites [6].

Cytarabine (cytosine arabinoside or arabinosyl cytosine, ara-C) (Figure 1) is a pyrimidine arabinoside nucleoside developed starting from spongothymidine or Ara-T. Produced synthetically in 1959 and patented in 1960, cytarabine entered the market in the USA in 1969 under the trade name Cytosar-U^®^ (Upjhon, Kalamazoo, MI, USA). Several cytarabine-based medicinal products are currently on the market in addition to Cytosar-U^®^, such as Aracytin^®^ (for pediatric use (Pfizer Italia, Latina, Italy) and Cytarabine Hospira^®^ (Hospira, Lake Forest, IL, USA) [9]. The cytotoxic activity of cytarabine is specific for cell cycle S phase after phosphorilation to cytosine arabinoside triphosphate (ara-CTP). It can inhibit DNA and RNA polymerases, blocking both DNA replication and transcription. Cytarabine is used for the treatment of acute myeloid leukemia (AML), acute lymphoblastic leukemia (ALL), and meningeal leukemia (lymphomatous meningitis), and in Hodgkin’s and non-Hodgkin’s lymphomas [10]. In AML, which is the mostly frequent leukemic form in adults, cytarabine is considered the most active drug. It is mostly associated with an antracycline drug, reaching about 70% of disease remission. On the contrary, no solid tumors are sensitive to cytarabine. Liposomal cytarabine was used in the intrathecal treatment of lymphomatous meningitis through the Depocyte^®^ (Almac Pharma Services Limited, Craigavon, UK) formulation approved by the EMA in 2001 [11] but withdrawn in 2006 after the concerns of the Committee for Medicinal Products for Human Use (CHMP) about the data presented by the Company, believed to be insufficient to reveal a greater efficacy/risk rate than intratecal methotrexate [12].

Nowaday, cytarabine is under evaluation (mostly associated with other anticancer drugs) in more than two hundred and fifty recruiting clinical studies (data from ClinicalTrials.gov database, update on 1 October 2021), of which seventy-one are phase III trials, mainly regarding AML, but also Langherans cell histiocytosis, T- cell lymphoma, mantle cell lymphoma, adult and pediatric acute lymphoblastic lymphoma and central nervous system (CNS) leukemia.

### 2.2. Vidarabine

In agreement with what is reported under cytarabine paragraph, the antimetabolite ability of arabinoside nucleosides may be useful also for the antiviral therapy. Vidarabine (arabinofuranosyl-adenine or adenine-arabinoside or ara-A) (Figure 1) is the first drug to become available in the USA for human severe *Herpes simplex* virus (HSV) infections. It is a synthetic analogue of the spongosine molecule spongouridine. Produced synthetically in 1960, it was initially studied as an anticancer drug and described as an antiviral in 1964 [13]. In 1976, the compound’s clinical antiviral utility was confirmed, specifically in the treatment of herpetic encephalitis and other herpetic infections that occasionally occur in infants [14]. In 1976, the first vidarabine-based product, Vira-A^®^ (King Pharmaceuticals, Bristol, TN, USA), ophthalmic 3% ointment, was approved by the FDA for acute kerato-conjunctivitis and recurrent epithelial keratitis caused by HSV type 1 and 2 (HSV-1 and -2). HSV-1 and 2 are two of the three human alpha *Herpes* viruses that cause infections worldwide. Like cytarabine, vidarabine is a prodrug, converted by viral kinases into adenine arabinoside triphosphate in infected cells. Once activated, the drug can act both as an inhibitor and as a substrate of viral DNA and RNA polymerases, interfering with DNA synthesis and transcription. When incorporated into a DNA strand, replacing many of the adenosine bases, vidarabine leads to a destabilisation of the newly synthesised DNA strand, as phosphodiester bridges can no longer be constructed [15]. Another proposed mechanism of action is the inhibition of pre-mRNA polyadenylation, catalysed by poly(A)-polymerase, by preventing the maturation of mRNA [16]. 

Although Vidarabine has covered an important role at the beginning of the antiviral therapy era, the advent of newer, more effective antivirals, such as acyclovir, azidothymidine (zidovudine) and others, has made vidarabine a drug no longer used in the USA and never used in Europe. King Pharmaceuticals discontinued the marketing of Vira-A^®^ ointment in June 2001 [17].

No recruiting trials with vidarabine are currently reported in ClinicalTrials.gov database (update in October 2021); although under the search keyword “vidarabine” many studies are shown, they are actually referred to the vidarabine derivative fludarabine.

### 2.3. Fludarabine

Fludarabine phosphate (arabinofuranosyl-2-fluoro-adenine-5-monophosphate) (Figure 1), a synthetic derivative of vidarabine, was designed to improve its in vivo anticancer activity.

Fludarabine monophosphate, differing from vidarabine for fluorine at position 2 on the pyrimidine structure and for mono-phosphorylation at 5’, is more resistant to the in vivo deamination than vidarabine which is, on the contrary, rapidly metabolized.

Once in the systemic circulation, fludarabine monophosphate is dephosphorylated to fludarabine within minutes and then phosphorylated to fludarabine triphosphate by deoxycytidine kinase upon entry into cells. Phosphorylated fludarabine has marked inhibitory activity on DNA polymerase and ribonucleotide reductase, and also a significant inhibitory activity on DNA-primase and RNA polymerase. On the whole, these actions produce a blockade of DNA and RNA synthesis [18,19].

In 1991, the FDA and in 1995 the EU granted marketing approval for Fludara^®^ (Genzyme Corporation, Cambridge, MA, USA) for the treatment of B-lineage chronic lymphatic leukaemia (CLL) in patients with sufficient bone marrow reserve. First-line treatment with fludarabine phosphate should only be initiated in patients with advanced disease, Rai stage III/IV (Binet stage C), or Rai stage I/II (Binet stage A/B) where the patient shows disease-related symptoms or disease progression. It is used as single drug or in combination with cyclophosphamide or mitoxantrone and dexamethasone or with the monoclonal antibody rituximab.

More than four-hundred recruiting trials (only twenty-eight in phase III) using fludarabine are registered on ClinicalTrials.gov database (update on 1 October 2021). Most of them are using the nucleoside in association with other drugs for hematological but also some solid cancer treatment.

### 2.4. Ziconotide

A very different drug from those above described is ziconotide (Figure 1), a peptide closely resembling the structure of the natural ω-conopeptide toxin discovered in the marine species *Conus Magus*, a gastropod of the Conidae family found in the Pacific Ocean. All species of the genus *Conus* are predators of small fish and are characterised by the production of neurotoxins used to immobilise the animals they feed on, thus compensating for the large difference in mobility between predator and prey. They are dangerous species also for humans, determining incidents of poisoning even fatal. The venom is contained in a gland inside a kind of stinger or “tooth”, which is ejected from the body by a very rapid movement [20]. 

The toxin of *Conus magus*, the ω-conotoxin MVIIA (ω-MVIIA), was discovered in the late 1960s and its therapeutic potential as an analgesic only emerged in the 1980s. It is similar to other ω-conotoxins, i.e., ω-GVIA, ω-MVIIC and ω-CVID, constituting a structurally related group of polypeptidic molecules binding with high affinity to voltage-gated calcium channels.

Ziconotide was produced synthetically by the Elan Corporation and approved by the FDA in 2004 and by the EMA in 2005 under the trade name Prialt^®^ (Riemser Pharma GmbH, Greifswald, Germany) [20]. 

Structurally, ziconotide is a peptide of twenty-five amino acids, six of which are cysteine residues that are linked in pairs by 3 disulfide bonds, which are necessary for the tertiary structure of the molecule relevant for its activity. Ziconotide analgesic effect is produced by the binding of the pore region of presynaptic N-type channel α_1B_ subunit and its physical occlusion [21,22]. The selective blockade of this channel in the spinal cord, with a consequent inhibition of the release of neurotransmitters and neuromodulators, such as glutamate, CGRP and substance P, involved in the spinal nociceptive stimulus transmission, explains its potent analgesic effect. 

It is indicated in the treatment of severe chronic pain, both oncological and secondary to other diseases, in patients refractory or intolerant to systemic analgesics or intrathecal morphine. Ziconotide can only be administered by an intrathecal infusion since it does not cross the blood-brain barrier [22].

Its main advantages are the potency of analgesia and the absence of spinal toxicity or respiratory depression as possible adverse events. In addition, it does not induce tolerance or habituation and does not cause physical or psychological dependence as, on the contrary, opioids do [20,22].

Currently, ziconotide is under evaluation in only one recruiting clinical trial for the treatment of neuropathic pain, to describe the methods used for intrathecal treatments containing this marine compound (ClinicalTrials.gov, update in October 2021).

### 2.5. Omega-3 Acid Ethyl Esters 

Polyunsaturated fatty acids (PUFAs) are relevant molecules for cellular metabolic processes. Among them, linoleic acid (LA) (ω-6) and α-linolenic acid (ALA) (ω-3) are essential fatty acids (EFAs) because they cannot be synthesised by humans or other higher animals. In the human body, they give rise to arachidonic acid (AA, ω-6), eicosapentaenoic acid (EPA, ω-3) (Figure 1) and docosahexaenoic acid (DHA, ω-3) (Figure 1), which play a key role in many cellular processes involved in regulation of body’s homeostasis. While AA gives rise to pro-inflammatory eicosanoids, the ω-3 EPA and DHA give rise to anti-inflammatory molecules. A balanced intake of ω-6/ ω-3 fatty acids is believed to be essential for proper functioning of physiological homeostatic mechanisms, and a higher consumption of ω-3 fatty acids (FAs) integrating the Mediterranean diet (ω-6 high) may protect against inflammation and related chronic diseases [23]. 

The marketed ω-3 FAs mostly derive from fishes belonging to families such as Engaulidae, Carangidae, Clupeidae, Osmeridae, Salmonidae and Scombroidae [3]. Recently, they have been produced also from fungus-like microorganisms named Thraustochytrids, high storers of ω-3 FAs [24]. 

Several studies associate ω-3 FAs with many beneficial properties for human health, including prevention of cardiovascular diseases such as thrombosis and atherosclerosis, protection of cognitive function in ageing, improvement of processes involved in child growth and prevention of depression [11,25,26]. 

Although frequently used as simple integrators, ω-3 PUFAs are also drugs approved in 2004 by the FDA and in 2005 by the EMA. In particular, their ethyl esters were approved for reducing plasma triglyceride levels in severe hypergtrygliceridemia [27]. In 2000, the EMA also granted approval for the prevention of relapses after a cardiac event, but more recently, after a review of the available studies, this indication was withdrawn [28].

Lovaza^®^ (GlaxoSmithKline, Research Triangle Park, NC, USA) is the first ω -3 FA-mediated drug to come onto the market. Subsequently, similar products such as Vascepa^®^ (Amarin Pharma, Bridgewater, NJ, USA), containing only EPA, and Epanova^®^ (AstraZeneca Pharmaceuticals, Wilmington, DE, USA) were approved by the FDA. In Europe, Eskim^®^ (Alfasigma, Bologna, Italy), Esapent^®^ (Pfizer Italia, Latina, Italy), Olevia^®^ (IBSA Farmaceutici Italia, Lodi, Italy) and a number of corresponding equivalents are now marketed [3]. 

Although the ability of ω-3 fatty acids to reduce circulating triglycerides is well established, the mechanism of action is still not well understood. Reduction of hepatic lipogenesis, cholesterol incorporation into VLDLs, cholesterol secretion and improved clearance of triglycerides from VLDL particles were suggested [29]. 

In addition to hypertrygliceridemia, other potential therapeutic applications of ω-3 PUFAs are under investigation to define the possible clinical use as drugs in further pathologies. Currently, about one hundred and fifty recruiting clinical trials are registered on ClinicalTrials.gov (update in October 2021), including six phase III trials. Among the recruiting phase III studies, ω-3 PUFAs are under evaluation for functional decline reduction in kidney transplanted subjects, for prevention of cognitive decline in old patients with subjective memory complaints or family history of Alzheimer’s disease and for recurrence of liver cancer after surgery. Interestingly, two phase-III recruiting studies are aimed at evaluating the efficacy and safety of ω-3 PUFAs in critical COVID-19 patients (ClinicalTrials.gov, update in October 2021).

### 2.6. Nelarabine

Nelarabine (arabinofuranosyl-6-methoxy-2-amino-purine) (Figure 1) is another synthetic spongosine derivative, structurally similar to vidarabine and fludarabine. It is the prodrug of guanosine arabinoside triphosphate (ara-GTP). Its development as anticancer drug derived from observations made on subjects with the purine nucleoside phosphorylase (PNP) deficiency, an autosomal recessive inherited disorder that causes a severe form of immunodeficiency since T lymphocytes undergo a selective accumulation of deoxyguanosine triphosphate causing a marked cytotoxic effect [30,31].

Nelarabine entered the market in the USA under the trade name Arranon^®^ in 2005, in the EU under the trade name Atriance^®^ (Novartis Europharm Limited, Dublin, UK) in 2007, for the treatment of patients with two rare cancers, T-cell acute lymphoblastic leukaemia (T-ALL) and T-cell lymphoblastic lymphoma (T-LBL), when the tumour does not respond, or has stopped responding, to at least two types of chemotherapy [32]. Once administered, nelarabine is rapidly converted to guanosine-arabinoside (ara-G) by the adenosine deaminase (ADA) and accumulates preferentially in T lymphoblasts. In these cells, the molecule is phosphorylated to ara-G triphosphate (ara-GTP), which acts as an antimetabolite of the guanosine nucleotide. The blockade of DNA synthesis can be achieved through the inhibition of various enzymes, i.e., DNA polymerase, DNA primase, DNA ligase and ribonucleotide reductase [30]. Nine clinical recruiting trials (four phase III) are currently using nelarabine in different protocols for treating newly diagnosed acute lymphoblastic leukemia or lymphoma (ClinicalTrials.gov, update in October 2021).

### 2.7. Trabectedin

Trabectedin, also called ecteinascidin 743 (ET-743) (Figure 1), was identified by PharmaMar researchers as the most abundant of a series of alkaloids isolated from *Ecteinascidia turbinata*, a sessile ascidian tunicate. Ascidians produce secondary metabolites, such as ecteinascidins, to protect themselves from predators. 

Receiving marketing authorisation from the EMA in 2007 and from the FDA in 2015, the anticancer drug trabectedin was marketed under the brand name Yondelis^®^ (PharmaMar, Colmenar Viejo, Spain) [33,34].

Trabectedin was obtained by total synthesis in 1996, but through a process that was too complex to meet the demand. Since the ascidia is not cultivable, the problem of supplying the molecule was solved by a semi-synthesis process starting from cyanosafracin B, a natural intermediate isolated from cultures of the bacterium *Pseudomonas fluorescens* [3]. The very potent anti-proliferative activity of trabectedin was firstly demonstrated in in vitro studies in different cell lines where the drug displayed an IC_50_ value in the very low range 0.0002–0.3 nM [35].

Trabectedin acts as an alkylating agent by binding to guanine residues located in the minor groove of the DNA double helix, thus blocking the activity of RNA polymerase II, and then the transcription of mRNA. Moreover, it demonstrated many other anticancer mechanisms. Another recognised mechanism is the specific inhibition of the MDR1 (Multi Drug Resistance 1) gene transcription, which codes for P-glycoprotein, a protein involved in cellular detoxification processes. In addition, trabectedin appears to impair the TC-NER (TransCription-Nucleotide Excision Repair) pathway, that has a role in repairing DNA lesions during the transcription process. Trabectedin is also able to reduce the inflammatory condition in the tumour microenvironment (TME), targeting monocytes and macrophages, which are characterised by an abnormal production of cytokines, and to inhibit angiogenesis [10,36]. 

The therapeutic indications approved are: (i) advanced (metastasised) soft tissue sarcoma when therapy with anthracyclines and ifosfamide is no longer effective or when these drugs cannot be given to patients; (ii) platinum-sensitive ovarian cancer recurrence after previous treatment (relapsed), in combination with pegylated liposomal doxorubicin [34]. Only thirteen recruiting studies are reported for trabectedin on ClinicalTrials.gov database (update in October 2021) and only one is in phase III, aimed at comparing the treatment of dedifferentiated liposarcoma with the MDM2 (murine double minute 2) antagonist milademetan vs. trabectedin.

### 2.8. Eribulin 

Eribulin (Figure 1) is a synthetic macrocyclic ketone analogue of halicondrin B, a high molecular weight compound isolated in 1986 from the *Halichondria okadai* sponge collected from the coast of Japan, and subsequently from other sponges of the Axinella, Phakellia and Lissodendoryx families. The production of this bioactive compound is indeed attributable to symbiotic dinoflagellate microalgae [6].

Eribulin showed potent cell growth inhibition with IC_50_ values between 0.09 and 9.5 nmol/L, in a heterogeneous variety of human cancer cell lines [37] and was approved as an antineoplastic medicinal product under the brand name Halaven^®^ (Eisai GmbH, Frankfurt am Main, Germany) since 2010 in the USA and 2011 in the EU.

It is approved for: (i) the treatment of adult patients with locally advanced or metastatic breast cancer undergoing disease progression after at least one chemotherapy regimen including the use of an anthracycline and a taxane; (ii) the treatment of adult patients with inoperable liposarcoma who already received anthracycline-containing therapy for advanced or metastatic disease. This latter indication was approved by the FDA and the EMA in 2016 [38,39]. 

The synthetic route of halicondrin B was determined by Yoshito Kishi and subsequent SAR (structure-activity relashionship) studies showing the macrocyclic ring in the molecule to be relevant for the anticancer activity [3,40].

Eribulin used as mesylate salt exerts its antineoplastic action as an antimitotic agent, blocking the polymerisation, and therefore the growth, of microtubules by binding to specific tubulin sites. Compared with other classical antimitotic drugs, Eribulin binding site on tubulin is different and its binding does not affect microtubule shortening [41]. This is important for the use of eribulin to treat cancers resistant to other microtubule blockers, such as taxanes.

New recruiting clinical trials (twenty-nine registered in ClinicalTrials.gov (update in October 2021), of which four are in phase III) are expected to give data about combination therapies containing eribulin mesylate in the treatment of metastatic breast cancer and other solid cancers as well. 

### 2.9. Brentuximab Vedotin

A great impulse in marine pharmacology has derived from the isolation of peptides named dolastatins from the mollusk *Dolabella auricolaria*, a marine snail found in the Indian Ocean. Nevertheless, dolastatins’ real producer was demonstrated to be the symbiotic cyanobacterium *Caldora penicillata*. Isolated dolastatins 10 and 15, with in vitro anti-mitotic activities inducing cell cycle arrest and apoptosis in tumour cell lines with subnanomolar IC_50,_ were the starting point for the synthesis of several derivatives after excessive adverse events observed with dolastatin-10 in human trials. The dolastatin 10-derivative auristatin was further modified to give monomethyl-auristatin E (MMAE), the first dolastatin-derived toxin to reach the market linked to a monoclonal antibody (Mab) for giving an antibody-drug conjugate (ADC) (Figure 2) [10,42,43]. 

Brentuximab vedotin is the active conjugate between MMAE and an anti-CD30 chimeric IgG_1_ Mab (brentuximab). CD30 is a surface marker belonging to the TNF superfamily, expressed in different lymphomas. In the ADC, an average of four MMAE groups are each linked to the Mab via a valine-citrulline (vc) linker to give vcMMAE (vedotin); MMAE groups become free into the cells for the activity of lysosome proteases [44,45].

The ADC was marketed as antineoplastic agent under the brand name Adcetris^®^ (Takeda Pharma A/S, Vallensbaek strand, Denmark) in 2011 in the USA and in 2012 in the EU [39,46], for the following clinical indications: (i) treatment of adult patients with relapsed or refractory CD30-positive Hodgkin’s lymphoma, in association with Doxorubicin, Vinblastine and Dacarbazine; (ii) in Hodgkin patients following autologous stem cell transplantation (ASCT), following at least two previous treatment regimens, when ASCT or polychemotherapy is not a therapeutic option; (iii) treatment of adult patients with relapsed or refractory systemic anaplastic large T-cell lymphoma (sALCL) [44].

Forty clinical trials are currently recruiting (two in phase III) exploring association of Brentuximab vedotin with other chemotherapeutic drugs for the treatment of lymphomas and also evaluating the ADC in solid cancers (melanoma and non-small cell lung cancer (NSCLC)) and non-cancer diseases (such as systemic sclerosis, mycosis fungoides, lymphomatoid papulosis, Sezary syndrome) (ClinicalTrials.gov, update in October 2021).

### 2.10. Lurbinectedin

Starting from trabectedin, described in paragraph 2.7, PharmaMar researchers synthesised the new anticancer agent lurbinectedin (PMO1183) (Figure 1), with a change in the C subunit of the molecule [47]. In particular, lurbinectedin is the tetrahydropyrrolo [4, 3, 2-de]quinolin-8(1H)-one analogue of trabectedin, maintaining similar mechanisms of action (in synthesis, inhibition of oncogenic genes’ transcription and TME modulation) but ameliorating the pharmacokinetic profile in cancer patients [47,48,49]. On June 2020, the FDA gave accelerated approval for lurbinectedin (Zepzelca™, Zepsyre^®^, PharmaMar, Colmenar Viejo, Spain) for the treatment of adult patients with metastatic small cell lung cancer (SCLC) under disease progression during or after platinum-based chemotherapy. SCLC is a rapidly progressive lethal type of cancer. The lurbinectedin approval was obtained thanks to the clinically meaningful effects and safety profile observed in a multicentre, open-label Phase II clinical trial in previously treated patients with advanced solid tumours [50,51]. Lurbinectedin has not yet been approved for use by the EMA, although it declared its orphan drug state in 2012 for ovary cancer and in 2019 for SCLC [52,53].

Very recently, the pharmacological profile of lurbinectedin and its clinical evaluation in SCLC has been reviewed by Rajput et al. [54].

The clinical use of lurbinectedin is currently under evaluation in further clinical trials of which five are recruiting (ClinicalTrials.gov, update in October 2021); in most of these studies, none of which are currently in phase III, the drug is evaluated in association with other drugs for the treatment of SCLC or other solid cancers.

### 2.11. Polatuzumab Vedotin

MMAE is used as component of other ADCs changing the targeting Mab (Figure 2). The second marketed MMAE-containing ADC is polatuzumab vedotin, where MAAE is conjugated to a humanized Mab directed against CD79b, a protein expressed in B lymphocytes, in chronic lymphatic leukemia and in most types of non-Hodgkin’s lymphomas. [55].

In 2019, the FDA granted accelerated market approval to polatuzumab vedotin (trade name Polivy™) in combination with bendamustine and rituximab for the treatment of diffuse large B-cell lymphoma under progression or failing to respond to two prior chemotherapy regimens. In 2020, it received a conditional marketing authorization also from the EMA [56,57].

The accelerated approval of polatuzumab vedotin is based on very positive and promising results from the phase IB/II study NCT02257567, where 40% of patients receiving the ADC achieved the primary outcome (complete response) versus 18% in the control arm. In addition, an improvement in median overall survival from 4.7 to 12.4 months was observed [58].

Several phase II trials are currently underway for the treatment of non-Hodgkin’s lymphoma, follicular lymphoma, and diffuse large cell lymphoma (BNCT01992653, NCT01691898, NCT02257567, NCT03677141, NCT02729896).

Moreover, the POLARIX (NCT03274492) study is currently underway to evaluate the efficacy, safety and pharmacokinetics of polatuzumab vedotin therapy in combination with R-CHP (rituximab, doxorubicin and prednisone and vincristine placebo) versus R-CHOP therapy (rituximab, cyclophosphamide, doxorubicin, vincristine and prednisone) in addition to polatuzumab vedotin placebo, in patients with untreated diffuse large B-cell lymphoma. This randomized study is carried out on 1000 patients and scheduled for completion in 2026. Lymphoma progression-free survival is the primary outcome, assessed at the onset of relapse or patient death up to 38 months [59]. Seventeen studies with polatuzumab vedotin are currently recruiting, of which three are in phase III, to evaluate new drug combinations containing this ADC for the treatment of large B-cell lymphoma and other lymphomas (ClinicalTrials.gov, update in October 2021).

### 2.12. Enfortumab Vedotin

Enfortumab vedotin is the third marketed ADC containing MMAE (Figure 2). The molecular target of the fully human Mab enfortumab is nectin-4, a transmembrane protein, part of a family of immunoglobulin-like adhesion molecules, expressed in many epithelial tumours, among which urothelial and breast carcinomas. Nectin-4 is relevant in carcinogenesis, promoting epithelial-mesenchymal transition (EMT), invasion and metastasis through different signalling pathways such as PI3K/Akt and Wnt/β-catenin [60]. 

In 2019 the FDA granted accelerated marketing authorisation to enfortumab vedotin (trade name Padcev™, Astellas Pharma Inc., Tokyo, Japan) for the treatment of adult patients with locally advanced urothelial carcinoma for which surgical removal or second-line therapy is not indicated [61]. The FDA approval was based on results from the phase III EV-201 study (NCT03474107), which demonstrated enfortumab vedotin to give a clinically meaningful response rate with a manageable and tolerable safety profile in patients with locally advanced or metastatic urothelial carcinoma who had been previously treated with platinum and anti-PD-1/L1 therapies [62]. 

The EV-301 study funded by Astellas Pharma Global Development (Northbrook, IL, USA) is ongoing to compare the efficacy of enfortumab vedotin to standard therapy as second line treatment in locally advanced or metastatic urothelial carcinoma patients. The randomised study, enrolling 608 volunteers, is composed of one group receiving enfortumab vedotin and another one receiving docetaxel, vinfluvin or paclitaxel. The study, which has survival at 36 months as the primary outcome, was scheduled for completion in February 2022 [63]. Nevertheless, in 2021, this drug has already received regular approval for the aforementioned use by the FDA and by the EMA.

Eleven recruiting studies (five in phase III) are reported in Clinical trials.gov database (update in October 2021) evaluating enfortumab vedotin in advanced urothelial cancer associated with other therapies, and also applied to other solid cancers treatment as single or combined drug. 

### 2.13. Belantamab Mafodotin

Among the auristatin derivatives, the new antimitotic monomethylauristatin F (MMAF) (Figure 2), was obtained introducing a charged C-terminal phenylalanine residue with respect to MMAE. MMAF is linked via the maleimido-caproyl spacer (mcMMAF, mafodotin) and also in this case, about four auristatin derivative molecules per single Mab entity is reached [64].

Belantamab mafodotin is the first marketed antibody (human Mab) that is conjugated to MMAF. The target of belantamab is B-cell maturation antigen (BCMA, CD269), which is highly expressed in malignant plasma cells in multiple myeloma (MM) [64].

On 5 August 2020, the FDA granted, also for this ADC, (trade name Blenrep^®^,produced by Glaxo Smith Kline, Dublin, UK), an accelerated approval for adult patients with relapsed or refractory MM who already received at least four prior therapies, including an anti-CD38 monoclonal antibody, a proteasome inhibitor and an immunomodulatory agent [65]. The EMA in 2017 granted the orphan drug status to Blenrep and in 2020 granted a conditional marketing authorisation for the same indications approved by the FDA [66].

The approval of belantamab mafodotin is based on positive data from the DREAMM-2 study (NCT 03525678), which enrolled 221 patients with relapsed/refractory MM who had received three or more prior lines of treatment. Belantamab mafodotin as monotherapy gave a clinically significant overall response rate (ORR) of 31% with the 2.5 mg/kg regimen [67]. Currently, nineteen recruiting trials (three phase III) are registered on ClinicalTrials.gov database (update in October 2021), evaluating different protocols comprehending belantamab mafodotin for MM or studying particular adverse events of this drug, such as corneal epithelial changes.

## 3. New Marine Drugs under Clinical Trial 

Among drugs already not marketed in the EU or USA, there are four drugs of marine origin, or with a marine component, undergoing phase III clinical trials while eight are in Phase II and twenty in Phase I (Table 2).

### 3.1. Phase III

Of the four marine drugs in phase III (Table 2), two are anticancer agents: plinabulin and morizomib.

Plinabulin (NPI-2358) (Figure 1), that is a diketopiperazine compound analogue of the natural compound phenilhistidinine (also called hamilide), derived from the fungus *Aspergillus ustus* blocks microtubule polymerization trough the colchicine-binding domain of β-tubulin [68] but also exerts immunostimulatory effects [69]; it is actually under investigation in an active not recruiting phase III trial combined with docetaxel for NSCLC (ClinicalTrials.gov, update in October 2021) therapy.

Marizomib (NPI-0052, salinosporamide A) (Figure 1), a β-Lactone-γ-lactam from the marine actinomycete *Salinispora tropica* that inhibits the ubiquitin-proteasome pathway [70], is under evaluation for the treatment of different cancers comprehending glioma, ependymoma, other solid cancers and also MM.

Tetrodotoxin and plitidepsin are the other two drugs under phase III trial (Figure 1).

Tetrodotoxin is an alkaloid from Pufferfish that is investigated for the analgesic properties in moderate-severe pain, since it blocks voltage-dependent sodium channels (VDSCs); 

Plitidepsin is a cyclic depsipeptide discovered in a sea tunicate *Aplidium albicans*, already marketed as Aplidin^®^ (PharmaMar, Colmenar Viejo, Spain) in Australia for the treatment of MM, but under investigation in phase III as anti-COVID agent, since its potent inhibition of the eukaryotic translation elongation factor eEF1A determines inhibition of viral replication [71].

### 3.2. Phases II 

Eight marine drugs are undergoing a phase II clinical trial (Table 2) and seven of them are investigated as anticancer agents. Among these latter, six are ADCs (Figure 2), containing antibodies recognising different antigenic targets, linked to marine-derived cytotoxic molecules. The marine toxin is the dolastatin analogue MMAE in five ADCs (tisotumab vedotin, ladiratuzumab vedotin, telisotuzumab vedotin, CAB-ROR2 and CX-2029) and a MMAE derivative (auristatin 0101) (Figure 2) in the drug W0101. CAB-ROR2 was produced with a new patented technology, named CAB (conditionally active biologics), in which the selected protein (in this case the Mab) have a function (in this case the antigen binding) that depends from the tissue environment. Another interesting technology was used for CX-2029, which is a particular ADC; it is indeed a probody drug conjugate (PDC) where MMAE is unmasked by tumor associated proteases [72].

At difference with the above cited phase II anticancer drugs, plocabulin (PM060184) is not an ADC but it is a *Lithoplocamia lithistoides* sponge-derived polyketide (Figure 1) which acts as a very potent antimitotic agent, binding to a specific β-tubulin site with possible activity on tumors resistant to other tubulin inhibitors [73]. 

The last drug under phase II is bryostatin, a macrocyclic polyketide (Figure 1) derived from *Bugula neritina* bryozoa and acting as potent inducer of protein kinase C (PKC). It was evaluated in the past as anticancer agent but currently is evaluated only for Alzheimer. The possible therapeutic utility in the neurodegenerative disease is due to the fact that activation of PKC induces the synthesis of proteins useful for neuroprotection and long-term memory consolidation processes [74]. 

### 3.3. Phase I

Twenty marine drugs are in the first phase of clinical investigation (Table 2) and seventeen of them are ADCs realized with dolastatin derivatives, i.e., MMAE, MMAF, auristatin 0101, auristatin F hydroxypropylamide (AF-HPA, XMT-1267), ambertstatin (A5269), the MMAF variant AGL-01250 and duostatin 3 (Figure 2). 

Among these ADCs, only STI-6129 is studied for therapy other than cancer, i.e., the rare disease light chain amiloidosis (AL), which actually has similarity with MM and sometimes is associated with this cancer. A further ADC, MORAb-202, is composed by the marine drug eribulin already marketed as unconjugated drug (see marketed drugs in this review), while in this case conjugated via a cathepsin cleavable linker to the humanized Mab farletuzumab, recognising the anti-folate receptor (FR)-α, overexpressed in platinum-resistant ovarian and other solid cancers. The results of the first phase I study in 22 patients with FR-*α* positive advanced solid cancer has been recently published suggesting the necessity of further investigation to clearly establish appropriate dosage and clinical utility of this ADC [75].

Another ADC, not containing a dolastatin derivative, is STRO-002. It contains the antimitotic tubulin binding compound taltobulin (SC209, HI286) (Figure 2), a synthetic analogue (3-aminophenyl derivative) of the *Hemiasterella minor* sponge-derived tripeptide hemiasterlin, conjugated to an another anti-FR-*α* Mab (human Mab SP8166) [76].

GTS-21 (Figure 1) is another drug under phase I for a non-cancer disease. It is a synthetic molecule derived from the alkaloid anabaseine, isolated from the marine intertidal *Amphiporus lactifloreus* worm. Its activity as agonist of α7 nicotinic receptors (AChRs α7) might be beneficial for obesity for which it is in phase I (ClinicalTrials.gov, update in October 2021), while in the past it was evaluated as drug for Alzheimer in a phase II study [77].

## 4. Conclusions 

**Figure 3:**
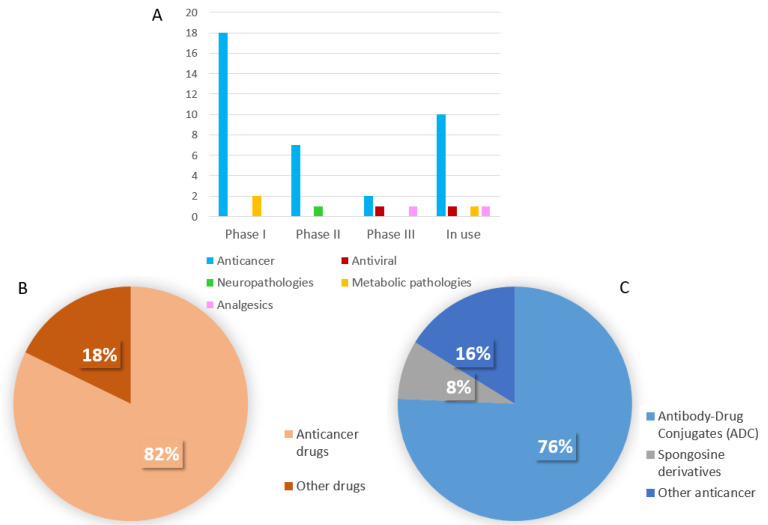
(**A**) Number of marine drugs under clinical trials and already marketed (in use), distributed for therapeutic class; (**B**) % distribution of all marine drugs (under clinical trials and already marketed) for their use (cancer vs. non cancer); (**C**) % distribution of marine anticancer agents (under clinical trials and already marketed) in different typology.

In the last ten years, the number of marine drugs on the EU and/or USA pharmaceutical market has duplicated. Moreover, a high increase of clinical trials has been registered, most of them currently in the first two phases (twenty-eight out of thirty-two). Most of the marine molecules both in clinical use and under trial are for the anticancer therapy (Figure 3A,B) and most of them are marine molecules conjugated to an antibody (ADCs) (Figure 3C).

Interestingly, while in the terrestrial environment the plant kingdom is the main source of pharmacologically active molecules, in the marine environment the animals currently are the prevalent source of marketed drugs. Usually, they are heterotrophic invertebrates with little or no mobility and living in symbiosis with microorganisms, which often are the real producers of secondary metabolites with relevance in drug discovery.

The ecological relationships and success of many sessile animals are based on secondary metabolism-derived toxic molecules used for predatory purposes, to paralyze high mobility prey, or to defend themselves from predators or inhibit the growth of other competing species. The marine habitats in which these animals evolved are in fact characterized by high competition in small living spaces. So, it is easy to find high cytotoxic agents with a potential antiproliferative and then anticancer activity. Spongosines, ecteinascidins, alicondrins, and dolastatins are marine toxins found to have very potent antiproliferative activity. They belong to different chemical structures suggesting that marine chemodiversity is largely explorable for new anticancer drug discovery. 

In addition to the antimetabolic spongosines and the DNA alkylating ecteinascidins, the other marine toxins used in the anticancer therapy display antimitotic activity by linking tubulin, suggesting this mechanism to be a widely used weapon for defensive/aggressive purposes by marine animals.

Although the high systemic human toxicity of marine toxins may be limiting in many cases their use as single agent, the ADCs strategy has been rapidly adapted to marine drugs, allowing the use of the potent marine toxins safely and effectively. Targeting the toxin to the site of action, much lower dosage ADCs can be used reaching therapeutic concentration in the target tissue and avoiding serious systemic adverse effects. 

Several variants of marine toxins and different chemical linkers between toxin and Mabs were prepared to obtain adequate pharmacokinetics. The linker has been important to optimize the toxin-mAb ratio, the stability of the conjugate in the bloodstream and the release of the toxin exclusively in the diseased tissue.

At present, the construction of conjugates in the form of ADCs is the main strategy adopted for delivering drugs of marine origin, but the conjugation with small molecules to obtain the so-called small molecule-drug conjugates (SMDCs) or the encapsulation in nanoparticles are other emerging strategies that may be relevant in the future for marine pharmacology. In conclusion, many future successes are expected from further marine chemistry exploration and from the use of biotechnological and nanotechnological strategies able to improve drug efficacy and/or safety.

## Figures and Tables

**Table 1:** Marketed drugs by the EMA and/or the FDA.

Generic Name	Brand Name/s	Date of Marketing Authorisation	Natural Source	Chemical Class	Clinical Use
Cytarabine	Cytosar-U Aracytin C.- Hospira	1969 (FDA)	Sponge	Nucleoside	Leukemia
Vidarabine	Vira-A	1976 (FDA)	Sponge	Nucleoside	Antiviral
Fludarabine	Fludara	1992 (FDA) 1994 (EMA)	Sponge	Nucleoside	Leukemia
Ziconotide	Prialt	2004 (FDA) 2005 (EMA)	Mollusk	Peptide	Chronic pain
Omega-3 acid ethyl esters	Lovaza (US) Eskim (EU) and others	2004 (FDA) 2005 (EMA)	Fish	PUFA	Hypertriglyceridemia
Nelarabine	Arranon (US) Atriance (EU)	2005 (FDA) 2007 (EMA)	Sponge	Nucleoside	Leukemia
Trabectedin	Yondelis	2007 (EMA) 2015 (FDA)	Tunicate	Alkaloid	Ovarian cancer, soft tissue sarcoma
Eribulin	Halaven	2010 (FDA)2011 (EMA)	Sponge	Macrolide	Breast cancer
Brentuximab vedotin	Adcetris	2011 (FDA) 2012 (EMA)	Mollusk/ cyanobacterium	ADC	Lymphomas
Lurbinectedin	Zepzelca	2020 (FDA)	Tunicate	Alkaloid	Ovarian cancer
Polatuzumab vedotin	Polivy	2019 (FDA) 2020 (EMA)	Mollusk/ cyanobacterium	ADC	Breast cancer
Enfortumab vedotin	Padcev	2019 (FDA) 2021 (EMA)	Mollusk/ cyanobacterium	ADC	Urothelial cancer
Belantamab mafodotin	Blenrep	2020 (FDA) 2020 (EMA)	Mollusk/ cyanobacterium	ADC	Multiple myeloma

ADC: Antibody Drug Conjugate; FDA: Food and Drug Administration; EMA: European Medicines Agency.

**Table 2:** Marine drugs under clinical trials subdivided into the three phases of clinical investigation.

Generic Name	Natural Source	Chemical Class	Clinical Use
**PHASE III**			
Tetrodotoxin	Pufferfish	Alkaloid	Chronic pain
Plinabulin	Fungus	Diketopiperazine	Cancer
Marizomib (salinosporamide A)	Actinomycetes	γ-lactam-β-lactone	Cancer
Plitidepsin	Tunicate	Depsipeptide	COVID-19
**PHASE II**			
Bryostatin	Bryozoan	Macrolide lactone	Alzheimer
Plocabulin (PM060184)	Sponge	Polyketide	Cancer
Tisotumab vedotin	Mollusk/cyanobacterium	ADC	Cancer
Ladiratuzumab vedotin (SGN-LIV1A)	Mollusk/cyanobacterium	ADC	Cancer
Telisotuzumab vedotin	Mollusk/cyanobacterium	ADC	Cancer
CAB-ROR2 (BA-3021)	Mollusk/cyanobacterium	ADC	Cancer
CX-2029	Mollusk/cyanobacterium	PDC	Cancer
W0101	Mollusk/cyanobacterium	ADC	Cancer
**PHASE I**			
GTS-21 (DMXBA)	Worm	Alkaloid	Obesity
Samrotamab vedotin	Mollusk/cyanobacterium	ADC	Cancer
Sirtratumab vedotin (ASG-15ME)	Mollusk/cyanobacterium	ADC	Cancer
SGN-CD48A	Mollusk/cyanobacterium	ADC	Cancer
ALT-P7	Mollusk/cyanobacterium	ADC	Cancer
ARX788	Mollusk/cyanobacterium	ADC	Cancer
Upifitamab rilsodotin (XMT-1536)	Mollusk/cyanobacterium	ADC	Cancer
AGS62P1	Mollusk/cyanobacterium	ADC	Cancer
PF-06804103	Mollusk/cyanobacterium	ADC	Cancer
Cofetuzumab pelidotin (ABBV-647)	Mollusk/cyanobacterium	ADC	Cancer
ZW-49	Mollusk/cyanobacterium	ADC	Cancer
MRG003	Mollusk/cyanobacterium	ADC	Cancer
STRO-002	Sponge	ADC	Cancer
MORAb-202	Sponge	ADC	Cancer
RC-88	Mollusk/cyanobacterium	ADC	Cancer
SGN-B6A	Mollusk/cyanobacterium	ADC	Cancer
SGN-CD228A	Mollusk/cyanobacterium	ADC	Cancer
FOR-46	Mollusk/cyanobacterium	ADC	Cancer
A-166	Mollusk/cyanobacterium	ADC	Cancer
STI-6129	Mollusk/cyanobacterium	ADC	Amyloidosis

ADC: Antibody Drug Conjugate; PDC: Probody Drug Conjugate.

## Data Availability

Not applicable.

## Abbreviation

**AA**: arachidonic acid	**FDA**: food and drug administration
**ADA**: adenosine deaminase	**HSV**: *Herpex symplex* virus
**ADC**: antibody-drug conjugate	**IgG**: immunoglobulin
**AL**: light chain amyloidosis	**LA**: linoleic acid
**ALA**: α-linolenic acid	**LBL**: lymphoblastic lymphoma
**sALCL**: systemic anaplastic large T-cell lymphoma	**Mab**: monoclonal antibody
**ALL**: acute lymphoblastic leukemia	**MDR**: multi drug resistance
**AML**: acute myeloid leukemia	**MDM2**: murine double minute 2
**Ara-A**: arabinofuranosyl-adenine, adenine-arabinoside, vidarabine	**MM**: multiple myeloma
**Ara-C**: cytosine arabinoside or arabinosyl cytosine or cytarabine	**MMAE**: monomethylauristatin E
**Ara-G**: guanosine-arabinoside	**MMAF**: monomethylauristatin F
**Ara-T**: arabinofuranosylthymine, spongothymidine	**NSCLC**: non-small cell lung cancer phosphorylase
**Ara-CTP**: cytosine arabinoside triphosphate	**PNP**: purine nucleoside
**Ara**-**GTP**: guanosine arabinoside triphosphate	**PDC**: probody drug conjugate
**ASCT**: autologous stem cell transplantation	**ORR**: overall response rate
**BCMA**: B-cell maturation antigen	**PUFA**: Polyunsaturated fatty acid
**CGRP**: calcitonin gene-related peptide	**sALCL**: systemic anaplastic large cell lymphoma
**CHMP**: committee for medicinal products for human use (EMA)	**SCLC**: small cell lung cancer
**CLL**: chronic lymphatic leukaemia	**SMDC**: small molecule drug conjugate
**CNS**: central nervous system	**T-ALL**: T-cell acute lymphoblastic leukaemia
**DHA**: docosahexaenoic acid	**T-LBL**: T-cell lymphoblastic lymphoma
**EFA**: essential fatty acid	**TC-NER**: Transcription-nucleotide excision repair
**EMT**: epithelial-mesenchymal transition	**TME**: tumor microenvironment
**EPA**: eicosapentaenoic acid	**TNF**: tumor necrosis factor
**EMA**: european medicine agency	**VDSC**: voltage-dependent sodium channel
**EU**: european Union	**VLDL**: very low density lipoprotein
**FA**: fatti acid	
